# Estimation of the Inhaled Dose of Pollutants in Different Micro-Environments: A Systematic Review of the Literature

**DOI:** 10.3390/toxics9060140

**Published:** 2021-06-12

**Authors:** Francesca Borghi, Andrea Spinazzè, Simone Mandaglio, Giacomo Fanti, Davide Campagnolo, Sabrina Rovelli, Marta Keller, Andrea Cattaneo, Domenico Maria Cavallo

**Affiliations:** Department of Science and High Technology, University of Insubria, 22100 Como, Italy; andrea.spinazze@uninsubria.it (A.S.); smandaglio@studenti.uninsubria.it (S.M.); giacomo.fanti@uninsubria.it (G.F.); davide.campagnolo@uninsubria.it (D.C.); sabrina.rovelli@uninsubria.it (S.R.); mkeller@uninsubria.it (M.K.); andrea.cattaneo@uninsubria.it (A.C.); domenico.cavallo@uninsubria.it (D.M.C.)

**Keywords:** personal exposure, pulmonary ventilation rate, residence time, indoor air pollution, outdoor air pollution, activity patterns

## Abstract

Recently, the need to assess personal exposure in different micro-environments has been highlighted. Further, estimating the inhaled dose of pollutants is considerably one of the most interesting parameters to be explored to complete the fundamental information obtained through exposure assessment, especially if associated with a dose-response approach. To analyze the main results obtained from the studies related to the estimation of the inhaled dose of pollutants in different micro-environments (environments in which an individual spends a part of his day), and to identify the influence of different parameters on it, a systematic review of the literature was performed. The principal outcomes from the considered studies outlined that (i) exposure concentration and residence time are among the most important parameters to be evaluated in the estimation of the inhaled dose, especially in transport environments. Further, (ii) the pulmonary ventilation rate can be of particular interest during active commuting because of its increase, which increases the inhalation of pollutants. From a methodological point of view, the advent of increasingly miniaturized, portable and low-cost technologies could favor these kinds of studies, both for the measurement of atmospheric pollutants and the real-time evaluation of physiological parameters used for estimation of the inhaled dose. The main results of this review also show some knowledge gaps. In particular, numerous studies have been conducted for the evaluation (in terms of personal exposure and estimation of the inhaled dose) of different PM fractions: other airborne pollutants, although harmful to human health, are less represented in studies of this type: for this reason, future studies should be conducted, also considering other air pollutants, not neglecting the assessment of exposure to PM. Moreover, many studies have been conducted indoors, where the population spends most of their daily time. However, it has been highlighted how particular environments, even if characterized by a shorter residence time, can contribute significantly to the dose of inhaled pollutants. These environments are, therefore, of particular importance and should be better evaluated in future studies, as well as occupational environments, where the work results in a high pulmonary ventilation rate. The attention of future studies should also be focused on these categories of subjects and occupational studies.

## 1. Introduction

Scientific literature has reported how people are continuously exposed to airborne pollutants from both indoor and outdoor sources, and the potential impact on health that results from this exposure. For these reasons, it is necessary to evaluate human exposure to these airborne contaminants.

As reported in the scientific literature, airborne pollutants may affect human health, especially in urban areas, representing hotspots of traffic emissions, both in indoor and outdoor environments. In particular, exposure to air pollutants in traffic environments has been related to long- and short-term cardiovascular and respiratory effects [1] from both epidemiological and toxicological studies [2]. It is also well known that during rush hours, commuters are usually exposed to high concentrations of traffic-related air pollutants [3], and that the rush hour period of commuting can potentially contribute to daily exposure, despite the commuting period representing a small portion of the daily period. It is recognized that the exposure measured in transportation environments may vary based on different parameters. For example, exposure may vary from inside to outside of private vehicles [4]. Moreover, personal exposure may also be affected by the road types, such as street configuration, road layout, meteorological conditions, and walking behavior of pedestrians [5], as reported, for example, by Correia and collaborators [6]. For these reasons, many studies have been conducted in several cities, considering different transit (as well as non-transit) environments, as well as assessing variation in exposure levels among commuting modes. As stated, many studies have focused on evaluating the exposure level.

The evaluation of exposure assessment in indoor environments have been widely represented in the scientific literature, because these MEs (e.g., home and office) play a crucial role in contributing to the total daily exposure and pollutant dose, due to the high proportion of time spent in indoor environments [7].

However, fewer studies were based on estimating the inhaled dose of pollutants. Inhaled dose can be estimated based on the (i) exposure concentration rate, (ii) time spent in a particular environment, and (iii) subject’s pulmonary ventilation rate. In particular, the pulmonary ventilation rate is often not considered in the experimental design of exposure assessment studies, probably due to technical and procedural limitations related to the measurement of this personal parameter, as well as some constraints such as discomfort in the investigated subjects [8]. As reported in [9], lack of personal data (i.e., age, weight, physical activity) makes dose estimation difficult, even though wearable technologies have become commercially available recently, making the measurement of airborne pollutant concentrations, for estimating the personal dose, technically more achievable.

The estimation of the inhaled dose may be of particular interest, especially in the transit environment, because of the different subject’s activity patterns and the different physical efforts (and subsequently different pulmonary ventilation rates) exerted in different transport modes. In particular, a higher pulmonary ventilation rate may be observed during active transportation (i.e., walking and cycling) [4,10]; as reported in the literature, a higher pulmonary ventilation rate can cause higher inhalation of pollutants.

Despite numerous exposure assessment studies reported in the literature, these have not been specifically analyzed in this work, because they are reported in other comprehensive reviews [2,11,12]. The principal aims of this review are to (i) identify the most important factors influencing the estimation of the inhaled dose of airborne pollutants (exposure concentration levels, subject pulmonary ventilation rate, and residence time in a particular micro-environment—MEs); (ii) report the results relating to the estimation of the inhaled dose obtained from the different studies analyzed, and (iii) highlight the gaps, in terms of pollutants, environments and subjects investigated in the paper, in order to direct future studies.

In particular, the paper identification process used for the systematic review of the literature is described in Section 2. The first presented results, relating to the (i) number of papers reported in this review, (ii) geolocation of the monitoring sites, (iii) type of pollutant, (iv) MEs, and (v) subjects investigated in the studies considered, are reported in Section 3. In Section 3.1, the methods used for estimating the inhaled dose are reported, while in Section 3.2, the main results related to the parameters necessary for estimating the inhaled dose (i.e., exposure concentration levels (Section 3.2.1), pulmonary ventilation rate (Section 3.2.2), and residence time (Section 3.2.3)) are presented. Finally, the results related to the inhaled dose of pollutants are reported in Section 3.3.1 (for indoor and outdoor MEs) and in Section 3.3.2 (for transit MEs).

## 2. Materials and Methods

This systematic review was conducted considering outcomes from three different databases (PubMed, Scopus, and ISI Web of Knowledge). For each database, a list of keywords, which was the same for the three databases, was arranged in a search query, even if the query structure was arranged as a function of the writing rules required by each selected database (Table 1).

**Figure 1:**
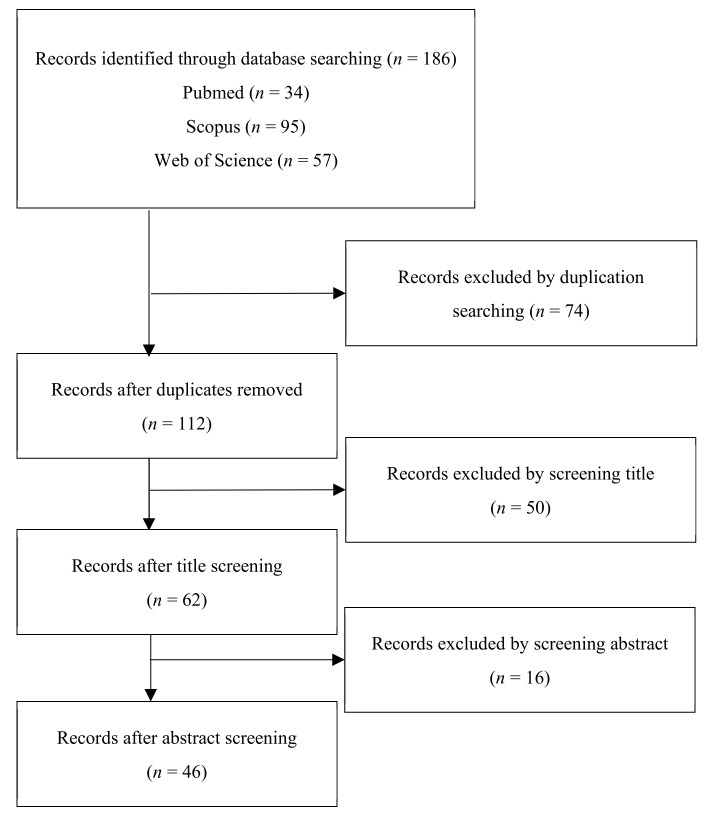
Flowchart of searched and reviewed literature (modified from [13]).

A total of 57 papers in the ISI Web of Knowledge, 34 papers in PubMed, and 95 papers in Scopus (last search: 11 February 2021) were found. Papers were detected and then selected following the chosen inclusion and exclusion criteria: only scientific papers written in the English language were considered in this review. In addition, only the articles that met the objectives of the review (or rather, analyzed the results concerning the dose of pollutants inhaled in different MEs) were considered. Exclusion criteria were case reports, conference papers, and publications that did not focus on the specific theme, or those published in languages other than English. Duplicates were removed from the total papers. After selecting in accordance with the aforementioned inclusion/exclusion criteria (done separately by three of the authors—F.B., G.F., and S.M.—in an attempt to reduce the operator error), 46 papers were found to be suitable for the present review. A flowchart of the literature research and review process (modified from [13]) is reported in Figure 1.

All the selected papers (*n* = 46) were independently reviewed by three of the authors (F.B., G.F., and S.M.), who selected papers that were relevant for review purposes in accordance with the inclusion criteria. The results of the eligible studies are described in the following sections, and then organized in paragraphs summarizing the findings of the studies and outlining their principal results.

## 3. Results

In Appendix A, the 46 articles considered in this systematic review are reported. As reported, the number of articles related to the topic of this review seemed to increase over the years (Table A1). This is probably caused by the advent of new technologies that allow the estimation of the inhaled dose of pollutants in real-time, and through the use of personal/individual monitors for (i) the measurement of pollutant concentration levels [14] and (ii) the measurement of physiological parameters, such as the pulmonary ventilation rate.

**Figure 2:**
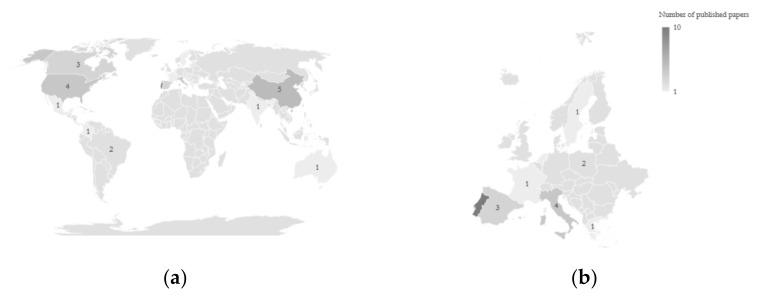
Number of studies performed by country at a global level (**a**) and in Europe (**b**).

As shown in Figure 2a,b, 27 articles were based on the monitoring performed in Europe (10 in Portugal, 4 in Italy, 3 in Spain, 2 each in Belgium, England and Poland, and 1 each in France, Greece, Holland and Sweden—Figure 2b) [1,6,8,10,15,16,17,18,19,20,21,22,23,24,25,26,27,28,29,30,31,32,33,34,35]; 4 studies were conducted in the USA [36,37,38,39], 3 in Canada [3,4,40], 2 in Brazil [41,42], and 1 each in Colombia [43] and Mexico [44]. Additionally, 5 studies were conducted in China [5,45,46,47,48], 1 each in Singapore [49], South Korea [50] and India [51]. Finally, only 1 study was conducted in Australia [9].

Although no exclusion criteria have been used regarding the kind of pollutant, most of the studies considered in this review investigated the particulate matter (PM) dose inhaled by the subjects under examination; as expected, focusing, for the majority of cases, on PM_2.5_ (27 articles), black carbon—BC (17 articles), and PM_10_ (16 articles). Numerous articles assessed ultrafine particles (UFPs) and PM_1_ (6 and 9 articles, respectively). Fewer articles estimated the inhaled doses of PM_0.5_, PM_4_, PM_5_, and total suspended particles (TSP) (1, 4, 1, and 1 articles, respectively). All the above studies considered the mass concentration as a metric for PM exposure; however, 3 additional articles were based on the measurement of the particle number concentration (other than the 6 articles concerning UFPs). Other than studies concerning the exposure to airborne particles, other studies focused on gaseous pollutants. Carbon monoxide (CO) was considered in 8 articles, nitrogen dioxide (NO_2_) in 4 articles, while carbon dioxide (CO_2_) as well as ozone (O_3_) were considered in 4 articles each. Few studies have investigated the inhaled dose of volatile organic compounds (VOCs) (4 articles), particle-bound polycyclic aromatic hydrocarbons (2 articles), formaldehyde (CH_2_O) (1 article), and aldehydes (1 article) (Table 2). The measurement of the above-mentioned pollutants was performed using both portable and personal instruments and fixed monitoring stations, as well as exposure estimation models.

The studies considered in this review focused on different MEs, defined as environments in which an individual spends a part of his day, both indoor and outdoor, as well as transport/transit MEs, often considered in the same study (Table 3). Indoor environments have not been described in detail in some cases [17,18,28,33,34,37,48], unlike other studies that have characterized environments such as home [18,19,20,29,30,42,50], dormitories [45,46], offices [45,46], schools or academies [29,31,50], laboratories [45,46], and hospitals [18]. Furthermore, some studies have been performed within fitness centers or gymnasiums [8,15,20,30], swimming pools [30], shops/supermarkets [30], cinemas/theaters [30], and restaurants or bars [30,50]. Similarly, some studies have been conducted in outdoor environments [18,34,41,48], focusing on particular environments such as beaches [30], playgrounds, gardens [30,50], or domestic outdoor environments [42]. Finally, numerous studies have been conducted on different public transport [3,21,42,48,50,52] or on private transport. In particular, several authors have focused on active transport: walking [4,5,10,17,18,19,20,21,23,24,27,28,30,38,43,44,49,51,52] and cycling [1,3,4,6,17,18,19,21,24,26,27,28,32,38,39,40,44,47,51,52].

Studies conducted on cars (private or taxis) have also been performed by several authors [1,3,6,17,18,19,20,24,26,28,30,32,38,44,49,51,52], as well as studies conducted on buses [1,6,10,18,19,24,26,30,32,38,43,44,49,51,52], metro [6,10,17,18,28,30,32,44,49], train [17,18,25,28,38], tram [18], motorcycle [32,51], and autorickshaw [51].

In the considered studies, different populations were monitored, and some cases composed of different subjects in heterogeneous groups (Table 4). In contrast, some authors focused on specific categories of selected subjects such as pregnant women [18], children [4,16,29,30,31,50], students (postgraduate and high school) [29,36,45,46,48], personnel and students in university [38], commuters [17,28,51,52], people in fitness centers [8,15,34], and elders [33,34].

### 3.1. Dose Estimation

Different formulas (chosen according to the aims and designs of the studies) have been used by the authors of the studies considered in this systematic review to estimate the inhaled dose of pollutants: in a mathematical form, pollutant inhalation depends on the pollutant concentration, the exposure time, and the minute ventilation (*VE*) (which depends on the subject’s physical effort, in addition to other parameters such as the subject’s physical condition, age, gender, etc.) are shown in Equation (1), as reported by different authors [5,6,10,16,24,29,31,35,38,42,44,49,50].

Pollutant _inhalation_[µg] = Pollutant _concentration_ [µg/m^3^] × *VE* [m^3^/min] × time [min] (1)

In the equations below, the units of measurement are not specified, as they can be expressed with different metrics, which are always consistent with each other (*D: dose; C: pollutant concentration; t: time; VE: minute ventilation; IR: inhalation rate; Vt: tidal volume; f: breathing frequency; BW: body weight*).

Similarly, the potential inhaled dose was estimated in the different MEs visited by the subject following Equation (2), multiplying the exposure in each ME by the time spent in the selected ME and by the subject inhalation rate (*IR*) [30].

(2) $$D=\overset{m}{\underset{j=1}{\sum }}\left(C_{j}\times t_{j}\times IR_{j}\right)$$

The estimation of the inhaled dose could consider other physiological parameters (Equation (3)), such as the tidal volume (*V_T_*) and the breathing frequencies (*f*), as reported by [23].

(3) $$D=V_{T}\times f\times C\times t$$

In some cases, the pulmonary ventilation rate of the subject was derived from the literature and adapted according to the intensity of the physical effort, during measurements [10,16,32,50]. In particular, some authors derived the pulmonary ventilation rate from the literature based on the metabolic equivalent of tasks done [4,9,24]. In other studies [8,17,47], the authors derived the subject’s ventilation rate by continuously monitoring the heart rate (HR) of the subject, using breaths per minute (bpm), and HR was used as a predictor for the ventilation rate [36].

To calculate the daily total inhaled dose, the partial doses estimated in different MEs were summed [16,36,40,43,50] or linearly extrapolated from the inhaled dose estimate on a 1 min time resolution [20]. As for the daily inhaled dose of pollutant, the annual inhaled dose could be estimated, as reported in [27].

The average inhaled dose in different MEs can also be estimated by integrating (i) the concentration of pollutants in different MEs, (ii) the subject inhalation rate, and (iii) the body weight (*BW*), as reported in Equation (4) [34].

(4) $$D=\frac{C\times VE}{BW}$$

Similarly, the assessment of the inhaled dose of pollutants can be performed using Equation (5) [8].

(5) $$D=\frac{C_{i}\times VE\times t}{BW}$$

Moreover, individual pollutant dose was estimated according to Equation (6), where *D_w_* is the dose of daily pollutant per unit body weight, *C_i_* is the personal pollutant concentration or daily pollutant concentration in the ME considered. *T_ij_* is the time spent per day for each person in activity intensity *j* in the considered MEs [46].

(6) $$D_{w}=\sum_{i=1}C_{i}\times \sum_{j=1}T_{ij}\times IR_{j}$$

The inhaled dose could also be estimated via transport mode and trip length [32,51], where *C*_i_ is the average concentration of the pollutants measured in one trip, *t* is the time spent in a round trip, *VE* is the minute ventilation, and km is the distance of the route (Equation (7)).

(7) $$Dose\;\left(µg/km\right)=\frac{C_{j}\times VE\times t}{km}$$

In addition to the equations used for the estimation of the inhaled dose reported above, more complex dosimetry models are reported in the literature. For example, Borghi and collaborators [28] used the MPPD (Multiple-Path Particle Dosimetry) [53,54] model, utilizing the Yeh-Shum symmetric model for humans. In particular, the MPPD model includes both human and rat respiratory tract models of the deposition and clearance of spherical particles [55]; the human model includes several deposition models, as well as the ICRP clearance model.

The information required by the human model are several and take into account parameters related to (i) airway morphometry; (ii) inhalant properties of the aerosol (e.g., density, diameter, aspect ratio); (iii) exposure conditions (e.g., breathing scenario, subject breathing frequencies, tidal volume) and (iv) deposition/clearance.

As reported in the literature [55], an evaluation of different human lung deposition models showed similar predictions of the total respiratory tract deposition fraction, as well as the deposition fractions in the tracheobronchial and alveolar regions. However, the most appropriate model (as well as the equations reported above) should be chosen according to (i) data availability and to (ii) the purpose of the study.

### 3.2. Principal Results—Parameters to Be Evaluated

Although this review was not carried out with the aim of analyzing the parameters that contribute to the estimation of the dose of inhaled pollutants, some main results obtained from the considered studies regarding (i) the exposure concentration levels, (ii) the pulmonary ventilation rate, and (iii) the residence time, are reported in the following paragraphs.

#### 3.2.1. Exposure Concentration Levels

Although the exposure concentration outcomes from the different studies were not comparable (because of the different study designs), the principal outcomes related to the concentration levels issue were reported. As stated before, most of the studies focused on PM, UFPs, and BC, and only a few of them focused on gaseous pollutants (CO, CO_2_, NO_2_, O_3_, VOCs, CH_2_O, and PAHs). As the exposure concentration pattern is similar among different pollutants, general results are reported below.

Faria et al. [30] suggested that a substantial fraction of particles was generated by indoor sources, showing indoor MEs as the main contributors to personal exposure to PM (and then to the respective inhaled dose). Similar results were found in another study [34], where particle concentration was found to be higher in school environments. Faria et al. [30] reported the average amount of children’s daily exposure to PM_2.5_ and PM_10_ in schools as 20.6 µg/m^3^ and 31.5 µg/m^3^, respectively. In particular, during weekdays, the classroom ME contributed to 42% and 50% of the PM_2.5_ and PM_10_ daily exposure, respectively. The outcomes of the study conducted by Carvalho et al. [42] showed a great variability in BC concentrations, exposure, and dose among the enrolled volunteers. In particular, the exposure may be different (up to 55%) for couples living together but working in different locations because of the different kinds of activities performed by the subjects and the time spent within the MEs considered, with transport being the category that contributed the most to the exposure and dose. In general, higher exposure levels were measured in transport MEs, while the lowest levels were measured at home and at work [19]. Among the transport MEs, trains presented lower PM_2.5_ and PM_10_ concentration levels than other MEs did, in the study conducted by Ramos et al. [10]. Therefore, the authors recommended trains, whenever possible, for daily commuting. Similarly, lower exposure concentration levels were measured in the train environment in other studies [17,19]. High pollutant concentrations were measured in the metro ME, probably due to the presence of emission sources in this environment (i.e., resuspension of particles due to turbulence and abrasion of rails, wheels, and brakes) [6,17]. Ramos et al. [32] suggested that car drivers and bus passengers in urban streets may be exposed to higher pollutant levels, compared with cyclists, even while commuting on the same streets. On the contrary, Velasco et al. [44] reported that commuting by cycling was the worst transport mode regarding exposure to different pollutants. Cyclists may be exposed to higher pollutant concentrations because of (i) passing aging or diesel vehicles, (ii) cycling through intersections and passing by bus stops, and (iii) mingling with motor vehicle traffic [47]. Finally, pedestrians generally presented low PM exposure concentrations [10].

#### 3.2.2. Pulmonary Ventilation Rate

The analysis of personal physiological parameters (i.e., pulmonary ventilation rate) for the estimation of the inhaled dose of pollutants can be of particular interest during active commuting. For instance, despite pedestrians presenting low pollutant (PM) concentration levels, VE increases as expected, subsequently increasing inhalation [10]. Similarly, cyclists seem to be exposed to lower pollutant concentration levels, than subjects who use the motorized mode of transport; however, due to the higher ventilation rates in cyclists, they presented the highest inhaled dose values [32]. In general, higher inhalation rates (as well as the commuting time) increased during active transportation [52] or during exercise [41].

#### 3.2.3. Residence Time

The amount of time spent in a particular environment is of great importance in estimating the inhaled dose of pollutants. As reported in a study [18], it was observed (via sensitivity analysis) that the most influential parameter in estimating the dose value is the time spent in a ME, followed by personal exposure concentration. Different studies showed, in particular, the importance of discriminating the average time spent by the subjects in indoor and outdoor environments. In this context, a large human activity pattern survey, such as the European EXPOLIS study [56] or the US NHAPS study [57] are certainly useful and widely used in dose estimation projects.

For example, Faria et al. [30] reported that children spent 86% of their time indoors, especially at home (55%) and in the classroom (27%). For these reasons, the authors stated that the risk assessment should focus on indoor MEs. Similar results were reported by other authors [48]. Regarding the commuting period, Dons et al. [19] evaluated that volunteers enrolled in their study spent 6.3% of their time (90 min per day) in transport MEs: in detail, the majority of trips were by car, but one-third of all travel time was by slow modes (cycling and walking). Other authors [32] indicated that the highest travel time was observed in their study for bus and bicycle transport modes. It is important to note that commuting time, as well as larger inhalation rates, contributed to the increase in the inhaled dose among active commuters [52].

### 3.3. Principal Results—Factors Influencing Inhaled Dose

#### 3.3.1. Indoor and Outdoor MEs

Faria et al. [30] demonstrated that indoor MEs are the main contributors to both personal exposure to PM and the respective inhaled dose. Similarly, 44% of the daily BC dose was estimated in indoor MEs (home and classroom) by Cunha-Lopes et al. [31], and these are the environments where subjects of the study (children) spent more than 80% of their time (home: 55%; classroom: 22%). On the contrary, Hu et al. [9] stated that the dose inhaled during indoor activities (i.e., sleeping, eating, working on campus, and doing home activities) is low, while the inhalation is high during outdoor activities (i.e., working, walking, and driving outdoors), although it should be considered that the relative importance of indoor and outdoor exposure concentration is highly dependent on location. Despite this, different authors have outlined how particular indoor activities may contribute significantly to the daily inhaled dose of pollutants. For example, cooking, as well as commuting period, may be considered as the main activity contributing to daily exposure [16]. Other authors [50] declared that the largest contribution to BC potential dose (41.7%) occurred in home environments because of the large amount of time spent there; however, it is important to note that the BC contribution for both exposure and potential dose is altered by different time-activity parameters (i.e., type of day, season, and gender). In particular, home activities, such as cooking and eating periods showed a high intensity level of potential BC dose (1.0), while sleeping presented a lower (0.5) level. Another study [30] showed contradictory results with that of Jeong and Park, where the contribution to the daily PM dose was higher while sleeping (weekdays: PM_2.5_: 16%, PM_10_: 13%; weekend: PM_2.5_: 36%, PM_10_: 32%), because of the sleeping duration (73%).

#### 3.3.2. Transit MEs

Regarding the transport MEs, in general, the inhaled dose during active transportation (i.e., walking and cycling) is higher than that inhaled in other passive and motorized transport modes, principally because of the higher ventilation rate and the time of residence associated with active transportation [6,17,38,44], even though Adams et al. [4] reported how the cycling trip dose value was significantly lower than that of the walking trip. In this particular case, the average dose values during the morning trip were 2.17 and 3.19 µg, respectively, for cycling and walking. During the afternoon trip, the trend was the same: the average dose values were 2.19 and 3.23 µg, respectively, for cycling and walking. Another study [1] reported how cyclists, compared to bus riders, inhaled 35% more PM (PM_1_ and PM_2.5_) and 62% more BC. Compared with car commuters, cyclists inhaled 50–90% more PM_1_ and PM_2.5_, and 48–84% more BC. Moreover, as reported by Dons et al. [19], the highest BC dose values were estimated for the bike-commuting mode (average dose of almost 200 ng/min). In detail, the dose ratios between different transport modes are the following: car/bicycle ratio = 0.41; car/walking ratio = 0.56; car/bus ratio = 0.82; and car/train ratio = 2.16. Similarly, other authors [21] stated that commuting to work by bicycle is associated with an increased long-term inhaled dose of BC. As anticipated, because of the heterogeneous study designs, it was difficult to compare the dose values obtained from the different studies analyzed in this review. Despite this, it is important to note that the exposure risk of the population in urban environments varies with population characteristics (i.e., age and gender). As reported by Qiu [5], adults had higher exposure concentrations (PM_2.5_, BC, and UFPs concentration) while walking, and teens had higher PM_2.5_ and BC inhaled doses than adults. This can be explained by the higher inhalation rates and longer trip durations associated with increased inhaled doses. For example, during weekdays, children spent 3.4% of their time commuting, and during this period, they inhaled 7.9% of their PM_2.5_ daily dose [30]. Moreover, children were intensely exposed to BC during commuting by diesel vehicles [50].

## 4. Discussion

In this systematic review, principal outcomes from 46 scientific papers related to the estimation of the inhaled dose of pollutants were summarized (Section 3).

The number of papers related to this topic (Table A1) seemed to increase in recent years, indicating an interest in this topic and the availability of technologies that allow the estimation of the inhaled dose more easily. The number of papers and studies related to this issue will likely continue to grow in the upcoming years, due to the advent of increasingly portable miniaturized and low-cost technologies. This could be valid both for the monitors used for the measurement of airborne pollutants, and for the technology used for the measurement of physiological parameters, useful for the estimation of the pollutant’s inhaled dose.

Most of the studies considered (N = 27) were conducted in Europe (Figure 2b), and the most investigated pollutants were PM2.5, BC, and PM10 (considered in 27, 17, and 16 papers, respectively) (Table 2). The high attention given to the measurement and evaluation of different PM fractions may be caused by the fact that PM is one of the most commonly investigated traffic-related pollutants, with respect to health effects (Morgenstern et al., 2007). Other gaseous pollutants considered in different studies are of interest, also because of their adverse effects on human health, and for this reason, future studies should try to fill this gap, focusing on other air pollutants other than PM.

The reviewed studies considered both indoor and outdoor MEs, as well as transport environments (Table 3). In particular, the most investigated indoor ME was the “home” environment (7 studies), probably because different MEs were considered over the course of the monitoring days, in different studies; for this reason, the ME “home” is often considered by studies characterized by a study design that provides a prolonged monitoring period. Another reason may be linked to the fact that the home environment is particularly interesting to evaluate (in terms of exposure and inhaled dose of pollutants) as much of the daily time is spent in this environment, both by adults and children.

Regarding transit environments, the “bicycle” and the “walking” modes were investigated in 20 and 19 studies, respectively. As mentioned, these are perhaps the most interesting MEs to evaluate, at least in regards to studies with the sole objective of estimating the inhaled dose to different pollutants, as subjects who commute walking or cycling generally result in higher inhaled pollutants’ doses, due to increased pulmonary ventilation rate.

In general, it would be interesting to analyze other MEs, where pulmonary ventilation rate can play a key role (e.g., during sports activities, or in occupational MEs, where workers are subjected to high physical efforts).

Both the general population and specifically selected populations were investigated, even though different studies aimed at the estimation of the pollutant dose inhaled by children and students (postgraduate and high school) (Table 4). The attention given to these subjects (children and students) is justified by the fact that numerous evidence in the scientific literature demonstrates that children at schools who are exposed to increased concentrations of air pollutants may have a higher risk for several health problems, including cognitive deficits. Moreover, children and students experience the greatest exposure in the school environment, and for these reasons, the evaluation of personal exposure and inhaled dose of children and students should continue to be performed, not neglecting other types of susceptible subjects (e.g., elderly, and subjects under high physical effort).

### 4.1. Parameters to Be Evaluated

Principal results related to the parameters to be evaluated in the estimation of the inhaled dose (i.e., exposure concentration levels, pulmonary ventilation rate, and residence time) (Section 3.2) show that exposure concentration levels may vary significantly across the different MEs considered, as well as among enrolled volunteers. In general, the authors stated that transport MEs contribute more (in terms of exposure levels) than non-transport MEs. Regarding the transport MEs, the train seems to present lower pollutant concentration levels. The metro environment seems to be characterized by the higher pollutant concentration levels, probably due to the presence of emission sources in this environment, such as the mechanical abrasion or rail/wheel, cleaning activities, surface air uptake from the surface, and wind erosion by intense air flow within the tunnels and platforms [58]. In addition, cycling may be characterized by high pollutant concentration levels, probably because cyclists are exposed directly (with no barriers) to the urban pollutants [10].

In general, due to the high variability of exposure concentrations in different MEs, (and also in ME of the same type, due to the different boundary conditions and because of the large number of factors that can influence the concentrations themselves), this parameter is of fundamental importance in estimating the inhaled dose. For the reasons listed above, it is also necessary to analyze the different emission sources and the various determinants of the exposure in selected MEs, preferably with high spatial and temporal resolution techniques, in order to highlight and analyze, for example, the presence of exposure peaks.

As expected, the analysis of personal physiological parameters (the pulmonary ventilation rate in particular) can be of particular interest during active commuting; in these cases, the VE increases, subsequently increasing the rate of pollutant inhalation. Finally, although the period of residence in a specific ME may vary according to the study design or the investigated population, this parameter is essential in the dose estimation process, especially for MEs for a prolonged period of time (usually indoor environments, such as homes, offices, and schools), although, as reported in the literature, MEs attended for a short period of time can also heavily affect the daily inhaled dose (e.g., transport MEs).

### 4.2. Factors Influencing Inhaled Dose

Some authors stated how indoor environments are those mainly contributing to the inhaled dose of pollutants because of the long period spent in these MEs (or because of particular activities performed indoors), but others have reported how higher inhaled dose value was estimated outdoors or in transit MEs. Obviously, the inhaled dose may vary according to the type of day (weekday/weekend), season, and gender, as well as the personal activity pattern, and subject physiological parameters.

Regarding the transport MEs, in general, the inhaled dose during active transportation (i.e., walking and cycling) is higher than that in passive and motorized transport modes, principally because higher ventilation rate and time of residence are associated with active transportation (Section 3.3). Results’ outcomes from the study considered in this review may be of particular interest, for example, to provide useful information to the general population, as well as to the authorities, to choose the most suitable means of transport (in terms of inhaled dose) and to redesign urban mobility.

As stated because of the heterogeneous study designs, it is difficult to compare the dose values obtained from the different studies analyzed in this review; however, it is important to note that the estimation of the inhaled dose of pollutant may vary according to different factors. For this reason, and thanks to the advent of increasingly miniaturized technologies, both for the direct-reading measurement of pollutant concentration at a personal level, and for the measurement of personal pulmonary ventilation rate, the estimation of the personal inhaled dose can be more easily done.

## 5. Conclusions

Despite the fact that studies aimed at the estimation of the inhaled dose of pollutants have increased in recent years, there remains an underlying problem relating to the use of this parameter—an aspect that was often not considered in the studies analyzed in this review. In addition, scientific literature concerning the estimation of the inhaled dose of pollutants are few, compared with the exposure assessment studies, perhaps due to the current evaluation paradigm. Actually, this paradigm only considers the external dose (exposure) and not the potential dose (i.e., the amount of contaminant inhaled, not all of which is actually absorbed), the applied dose (i.e., the amount of contaminant at the absorption barrier that can be absorbed by the body), the internal dose (i.e., the amount of contaminant that passes the exchange boundary and into the blood, or the amount of the contaminant that can interact with organs and tissues to cause biological effects), nor the biologically-effective dose (i.e., the amount of contaminant that interacts with the internal target tissue or organ). Furthermore, in the risk assessment approach, the intensity of the activity performed (e.g., lung ventilation rate) is not considered for a correction of the measured exposure concentrations.

Dosimetry (i.e., measurement or estimation of the internal dose of pollutants) can be useful, especially in exposure assessment studies because (i) it provides useful information regarding the relationship between exposure concentration and biological response and (ii) it can improve the accuracy of risk assessment, reducing the uncertainty, providing reliable estimates of the internal dose at the target tissue [55]. For these reasons, dosimetry models and estimations are currently used in risk assessment studies and in various applications (e.g., for the evaluation of dose distribution in an exposed population, including a sensitive subpopulation). Moreover, the study of [44], showed that using exposure concentration instead of inhaled dose may cause bias interpretation in the health risks associated to different transport modes. Therefore, Velasco et al. [44] suggested that a final assessment should focus on inhaled dose instead of exposure concentrations. The estimation of the inhaled dose while commuting may be of particular interest in active commuting (e.g., walking and cycling), with physical effort being considered. As discussed in this work and as reported in a study [4], a dose-based exposure approach includes the inhalation rate of an individual, to account for changes in exposure due to increased or decreased energy expenditure. Therefore, considering the estimation of the inhaled dose of pollutants is particularly important when comparing air pollution exposure between active transportation modes, because of the higher inhalation rate. Another issue that must be considered is the fact that the mechanisms between air pollution and pulmonary response may be more sensitive to the inhaled dose of air pollutants rather than to the sole pollutant exposure levels [36]. Evaluating the outcomes from inhaled dose estimations allows the investigation of possible interactions between physical activity and air pollution to analyze the respiratory effects of these factors appropriately.

As reported, many studies have focused on particular aspects—numerous studies have been conducted for the evaluation (in terms of personal exposure and estimation of the inhaled dose) of different PM fractions; other airborne pollutants, although harmful to human health, are less represented in studies of this type, and for this reason, future studies should be conducted considering other air pollutants, not neglecting the assessment of exposure to PM. Moreover, many studies have been conducted indoors, where the population spends most of their daily time. However, it has been highlighted how particular MEs, even if characterized by a shorter residence time (e.g., commuting and transit environments) can contribute significantly to the dose of inhaled pollutants; these environments are therefore of particular importance and should be better evaluated. Other environments poorly represented in this context are occupational environments, where the worker results in high pulmonary ventilation rate; the attention of future studies should also be focused on these categories of subjects and on occupational studies.

In conclusion, the analyzed studies revealed that the estimation of the inhaled dose is interesting to evaluate in particular environments/contexts, such as transit environments (especially with reference to active commuting); in these environments, therefore, more in-depth evaluations (in terms of pollutants investigated) could be carried out. It could also be useful to deepen the issue of the estimation of the inhaled dose in occupational environments, or in other conditions where the subject experiences a high pulmonary ventilation rate.

## Figures and Tables

**Table 1:** Queries used for the search in the three different databases.

Database	Search Query
PubMed	(((((((((((((micro-environment*) OR transport*) OR mode) OR commuting) OR car*) OR bus*) OR public) OR subway) OR underground) OR cyclist*) OR train*)) AND “inhaled dose”) AND ((pollut*) OR “air pollution”)
Scopus	TITLE-ABS-KEY (micro-environment* OR transport* OR mode OR commuting OR car* OR bus* OR public OR subway OR underground OR cyclist* OR train*) AND TITLE-ABS-KEY (“inhaled dose”) AND TITLE-ABS-KEY (pollut* OR “air pollution”)
Web of Science	TS = (micro-environment* OR transport* OR mode OR commuting OR car* OR bus* OR public OR subway OR underground OR cyclist* OR train*) AND TS = (“inhaled dose”) AND TS = (pollut* OR “air pollution”)

**Table 2:** Pollutants considered in the studies under review. UFPs: ultrafine particles (particle number); TSP: total suspended particles; BC: black carbon; VOCs: volatile organic compounds; PAHs: polycyclic aromatic hydrocarbons.

Pollutant	Number of Papers	Reference
UFPs	6	[5,16,17,23,24,47]
PM_0.5_	1	[8]
PM_1_	8	[1,8,15,17,18,23,28,32,47,51]
PM_2.5_	26	[1,4,5,6,8,10,17,18,22,23,24,26,28,31,32,36,38,40,41,43,44,45,46,47,48,49,51,52]
PM_4_	4	[15,17,18,28,32]
PM_5_	1	[8]
PM_10_	16	[6,8,10,17,18,22,23,26,28,31,32,33,34,35,40,41,47]
TSP	2	[17,18]
BC	16	[1,5,6,16,19,20,21,24,31,36,42,43,48,49,50,52]
Particle number	3	[1,26,49]
CO	7	[8,9,24,32,43,49,52]
CO_2_	4	[8,15,24,32]
NO_2_	5	[3,17,18,27,52]
O_3_	4	[8,15,32,36]
VOCs	3	[8,15,29,32]
CH_2_O	1	[8]
Aldehydes	1	[29]
Particle-bound PAHs	1	[49]

**Table 3:** MEs considered in the studies reviewed.

ME	Number of Papers	Reference
Indoor (general)	2	[17,18,28,33,34,37,48]
Home	7	[18,19,20,29,30,42,50]
Dormitories	2	[45,46]
Offices	2	[45,46]
Schools or academies	3	[29,31,50]
Laboratories	2	[45,46]
Hospitals	1	[18]
Fitness centers or gymnasiums	4	[8,15,20,30]
Swimming pools	1	[30]
Shops/Supermarkets	1	[30]
Cinemas/Theaters	1	[30]
Restaurants/Bars	2	[30,50]
Outdoor (general)	4	[18,34,41,48]
Beaches	1	[30]
Playgrounds/Gardens	2	[30,50]
Domestic outdoor	1	[42]
Public transport (general)	6	[3,21,42,48,50,52]
Walking	19	[4,5,27,28,30,38,43,44,49,51,52,10,17,18,19,20,21,23,24]
Bicycle	20	[1,3,4,6,17,18,19,21,24,26,27,28,32,38,39,40,44,47,51,52]
Car	17	[1,3,6,17,18,19,20,24,26,28,30,32,38,44,49,51,52]
Bus	15	[1,6,10,18,19,24,26,30,32,38,43,44,49,51,52]
Metro	9	[6,10,17,18,28,30,32,44,49]
Train	5	[17,18,25,28,38]
Tram	1	[18]
Motorcycle	2	[32,51]
Autorickshaw	1	[51]

**Table 4:** Categories of subjects considered in this review.

Subject	Number of Papers	Reference
Pregnant	1	[18]
Children	5	[4,16,29,30,31,50]
Students (postgraduate and high school)	5	[29,36,45,46,48]
Personnel and students in university	1	[38]
Commuters	4	[17,28,51,52]
People in fitness centers	3	[8,15,34]
Elders	2	[33,34]

## Data Availability

Not applicable.

## Appendix A

**Table A1:** Articles considered in this systematic review.

Authors	Title	Year of Publication
Dua and Hopke	Hygroscopicity of indoor aerosols and its influence on the deposition of inhaled radon decay products	1995
Abadie et al.	Particle pollution in the French high-speed train (TGV) smoker cars: measurement and prediction of passengers’ exposure	2004
Zuurbier et al.	In-traffic air pollution exposure and CC16, blood coagulation, and inflammation markers in healthy adults	2011
de Nazelle et al.	A travel mode comparison of commuters’ exposures to air pollutants in Barcelona	2012
Dons et al.	Personal exposure to black carbon in transport microenvironments	2012
Nwokoro et al.	Cycling to work in London and inhaled dose of black carbon	2012
Buonanno et al.	Children’s exposure assessment to ultrafine particles and black carbon: The role of transport and cooking activities	2013
Almeida et al.	Exposure and dose assessment to particle components among an elderly population	2014
Faria et al.	Evaluation of a numerical methodology to estimate pedestrians’ energy consumption and PM inhalation	2014
Hu et al.	Air pollution exposure estimation and finding association with human activity using wearable sensor network	2014
Vouitsis et al.	Microenvironment particle measurements in Thessaloniki, Greece	2014
Almeida et al.	Exposure and inhaled dose of susceptible population to chemical elements in atmospheric particles	2015
Ramos et al.	Estimating the inhaled dose of pollutants during indoor physical activity	2015
Ramos et al.	Comparison of particulate matter inhalation for users of different transport modes in Lisbon	2015
Adams et al.	Air pollution exposure: An activity pattern approach for active transportation	2016
Cepeda et al.	Levels of ambient air pollution according to mode of transport: a systematic review	2016
Lei et al.	Individual exposure of graduate students to PM2.5 and black carbon in Shanghai, China	2016
Pasalic et al.	Air pollution, physical activity, and markers of acute airway oxidative stress and inflammation in adolescents.	2016
Ramos et al.	Air pollutant exposure and inhaled dose during urban commuting: a comparison between cycling and motorized modes	2016
Zwozdziak et al.	Implications of the aerosol size distribution modal structure of trace and major elements on human exposure, inhaled dose and relevance to the PM2.5 and PM10 metrics in a European pollution hotspot urban area	2016
Broach and Bigazzi	Existence and use of low-pollution route options for observed bicycling trips	2017
Chaney et al.	Personal exposure to fine particulate air pollution while commuting: An examination of six transport modes on an urban arterial roadway	2017
Dons et al.	Wearable sensors for personal monitoring and estimation of inhaled traffic-related air pollution: evaluation of methods	2017
Jeong and Park	Contribution of time-activity pattern and microenvironment to black carbon (BC) inhalation exposure and potential internal dose among elementary school children	2017
Tan et al.	Particle exposure and inhaled dose during commuting in Singapore	2017
Apparicio et al.	Exposure to noise and air pollution by mode of transportation during rush T hours in Montreal	2018
Carvalho et al.	Variations in individuals’ exposure to black carbon particles during their daily activities: A screening study in Brazil	2018
Pasqua et al.	Exercising in air pollution: The cleanest versus dirtiest Cities challenge	2018
Slezakova et al.	Indoor air quality in health clubs: Impact of occupancy and type of performed activities on exposure levels	2018
Xu et al.	Estimated individual inhaled dose of fine particles and indicators of lung function: A pilot study among Chinese young adults.	2018
Betancourt et al.	Personal exposure to air pollutants in a Bus Rapid Transit System: Impact of fleet age and emission standard	2019
Borghi et al.	Evaluation of the inhaled dose across different microenvironments	2019
Correia et al.	Particle exposure and inhaled dose while commuting in Lisbon	2019
Cunha-Lopes et al.	Children’s exposure to sized-fractioned particulate matter and black carbon in an urban environment	2019
Engström and Forsberg	Health impacts of active commuters’ exposure to traffic-related air T pollution in Stockholm, Sweden	2019
Li et al.	Associations between inhaled doses of PM2.5-bound polycyclic aromatic hydrocarbons and fractional exhaled nitric oxide	2019
Polednik and Piotrowicz	Pedestrian exposure to traffic-related particles along a city road in Lublin, Poland	2019
Qiu et al.	Exposure assessment of cyclists to UFP and PM on urban routes in Xi’an, China	2019
Qiu et al.	Pedestrian exposure to PM2.5, BC and UFP of adults and teens: A case study in Xi’an, China	2019
Velasco et al.	Particle exposure and inhaled dose while commuting by public transport in Mexico City	2019
Borghi et al.	Commuters’ personal exposure assessment and evaluation of inhaled dose to different atmospheric pollutants	2020
Borghi et al.	Estimation of the inhaled dose of airborne pollutants during commuting: Case study and application for the general population	2020
Buregeya et al.	Short-term impact of traffic-related particulate matter and noise exposure on cardiac function	2020
Faria et al.	Children’s exposure and dose assessment to particulate matter in Lisbon	2020
Lizana et al.	Contribution of indoor microenvironments to the daily inhaled dose of air pollutants in children: The importance of bedrooms	2020
Manojkumar, Monishraj and Srimuruganandam	Commuter exposure concentrations and inhalation doses in traffic and residential routes of Vellore city, India	2021

## References

[1] Vouitsis I., Taimisto P., Kelessis A., Samaras Z. (2014). Microenvironment particle measurements in Thessaloniki, Greece. Urban Clim..

[2] Karanasiou A., Viana M., Querol X., Moreno T., de Leeuw F. (2014). Assessment of personal exposure to particulate air pollution during commuting in European cities-Recommendations and policy implications. Sci. Total Environ..

[3] Apparicio P., Gelb J., Carrier M., Mathieu M.È., Kingham S. (2018). Exposure to noise and air pollution by mode of transportation during rush hours in Montreal. J. Transp. Geogr..

[4] Adams M.D., Yiannakoulias N., Kanaroglou P.S. (2016). Air pollution exposure: An activity pattern approach for active transportation. Atmos. Environ..

[5] Qiu Z., Lv H., Zhang F., Wang W., Hao Y. (2019). Pedestrian exposure to PM2.5, BC and UFP of adults and teens: A case study in Xi’an, China. Sustain. Cities Soc..

[6] Correia C., Martins V., Cunha-Lopes I., Faria T., Diapouli E., Eleftheriadis K., Almeida S.M. (2020). Particle exposure and inhaled dose while commuting in Lisbon. Environ. Pollut..

[7] Cattaneo A., Campo L., Iodice S., Spinazzè A., Olgiati L., Borghi F., Polledri E., Angelici L., Cavallo D.M., Fustinoni S. (2021). Environmental and biological monitoring of personal exposure to air pollutants of adult people living in a metropolitan area. Sci. Total Environ..

[8] Ramos C.A., Reis J.F., Almeida T., Alves F., Wolterbeek H.T., Almeida S.M. (2015). Estimating the inhaled dose of pollutants during indoor physical activity. Sci. Total Environ.

[9] Hu K., Davison T., Rahman A., Sivaraman V. Air pollution exposure estimation and finding association with human activity using wearable sensor network. Proceedings of the MLSDA 2014 2nd Workshop on Machine Learning for Sensory Data Analysis—MLSDA’14.

[10] Ramos M.J., Vasconcelos A., Faria M. (2015). Comparison of particulate matter inhalation for users of different transport modes in Lisbon. Transp. Res. Procedia.

[11] Monn C. (2001). Exposure assessment of air pollutants: A review on spatial heterogeneity and indoor/outdoor/personal exposure to suspended particulate matter, nitrogen dioxide and ozone. Atmos. Environ..

[12] Han X., Naeher L.P. (2006). A review of traffic-related air pollution exposure assessment studies in the developing world. Environ. Int..

[13] Moher D., Liberati A., Tetzlaff J., Altman D.G., Altman D., Antes G., Atkins D., Barbour V., Barrowman N., Berlin J.A. (2009). Preferred reporting items for systematic reviews and meta-analyses: The PRISMA statement. PLoS Med..

[14] Borghi F., Spinazzè A., Rovelli S., Campagnolo D., Del Buono L., Cattaneo A., Cavallo D.M. (2017). Miniaturized monitors for assessment of exposure to air pollutants: A review. Int. J. Environ. Res. Public Health.

[15] Slezakova K., Peixoto C., Pereira M.d.C., Morais S. (2018). Indoor air quality in health clubs: Impact of occupancy and type of performed activities on exposure levels. J. Hazard. Mater..

[16] Buonanno G., Stabile L., Morawska L., Russi A. (2013). Children exposure assessment to ultrafine particles and black carbon: The role of transport and cooking activities. Atmos. Environ..

[17] Borghi F., Spinazzè A., Fanti G., Campagnolo D., Rovelli S., Keller M., Cattaneo A., Cavallo D.M. (2020). Commuters’ personal exposure assessment and evaluation of inhaled dose to different atmospheric pollutants. Int. J. Environ. Res. Public Health.

[18] Borghi F., Cattaneo A., Spinazzè A., Manno A., Rovelli S., Campagnolo D., Vicenzi M., Mariani J., Bollati V., Cavallo D.M. (2019). Evaluation of the inhaled dose across different microenvironments. Proceedings of the IOP Conference Series: Earth and Environmental Science.

[19] Dons E., Int Panis L., Van Poppel M., Theunis J., Wets G. (2012). Personal exposure to Black Carbon in transport microenvironments. Atmos. Environ..

[20] Dons E., Laeremans M., Orjuela J.P., Avila-Palencia I., Carrasco-Turigas G., Cole-Hunter T., Anaya-Boig E., Standaert A., De Boever P., Nawrot T. (2017). Wearable Sensors for Personal Monitoring and Estimation of Inhaled Traffic-Related Air Pollution: Evaluation of Methods. Environ. Sci. Technol..

[21] Nwokoro C., Ewin C., Harrison C., Ibrahim M., Dundas I., Dickson I., Mushtaq N., Grigg J. (2012). Cycling to work in London and inhaled dose of black carbon. Eur. Respir. J..

[22] Zwozdziak A., Gini M.I., Samek L., Rogula-Kozlowska W., Sowka I., Eleftheriadis K. (2017). Implications of the aerosol size distribution modal structure of trace and major elements on human exposure, inhaled dose and relevance to the PM2.5 and PM10 metrics in a European pollution hotspot urban area. J. Aerosol Sci..

[23] Polednik B., Piotrowicz A. (2020). Pedestrian exposure to traffic-related particles along a city road in Lublin, Poland. Atmos. Pollut. Res..

[24] De Nazelle A., Fruin S., Westerdahl D., Martinez D., Ripoll A., Kubesch N., Nieuwenhuijsen M. (2012). A travel mode comparison of commuters’ exposures to air pollutants in Barcelona. Atmos. Environ..

[25] Abadie M., Limam K., Bouilly J., Génin D. (2004). Particle pollution in the French high-speed train (TGV) smoker cars: Measurement and prediction of passengers exposure. Atmos. Environ..

[26] Zuurbier M., Hoek G., Oldenwening M., Meliefste K., Krop E., van den Hazel P., Brunekreef B. (2011). In-traffic air pollution exposure and CC16, blood coagulation, and inflammation markers in healthy adults. Environ. Health Perspect..

[27] Engström E., Forsberg B. (2019). Health impacts of active commuters’ exposure to traffic-related air pollution in Stockholm, Sweden. J. Transp. Health.

[28] Borghi F., Fanti G., Cattaneo A., Campagnolo D., Rovelli S., Keller M., Spinazz A., Cavallo D.M. (2020). Estimation of the Inhaled Dose of Airborne Pollutants during Commuting: Case Study and Application for the General Population. Int. J. Environ. Res. Public Health.

[29] Lizana J., Almeida S.M., Serrano-Jiménez A., Becerra J.A., Gil-Báez M., Barrios-Padura A., Chacartegui R. (2020). Contribution of indoor microenvironments to the daily inhaled dose of air pollutants in children. The importance of bedrooms. The importance of bedrooms. Build. Environ..

[30] Faria T., Martins V., Correia C., Canha N., Diapouli E., Manousakas M., Eleftheriadis K., Almeida S.M. (2020). Children’s exposure and dose assessment to particulate matter in Lisbon. Build. Environ..

[31] Cunha-Lopes I., Martins V., Faria T., Correia C., Almeida S.M. (2019). Children’s exposure to sized-fractioned particulate matter and black carbon in an urban environment. Build. Environ..

[32] Ramos C.A., Wolterbeek H.T., Almeida S.M. (2016). Air pollutant exposure and inhaled dose during urban commuting: A comparison between cycling and motorized modes. Air Qual. Atmos. Health.

[33] Almeida-Silva M., Almeida S.M., Pegas P.N., Nunes T., Alves C.A., Wolterbeek H.T. (2015). Exposure and dose assessment to particle components among an elderly population. Atmos. Environ..

[34] Almeida S.M., Ramos C.A., Almeida-Silva M. (2016). Exposure and inhaled dose of susceptible population to chemical elements in atmospheric particles. J. Radioanal. Nucl. Chem..

[35] Faria M., Duarte G., Vasconcelos A., Farias T. (2014). Evaluation of a numerical methodology to estimate pedestrians’ energy consumption and PM inhalation. Transp. Res. Procedia.

[36] Pasalic E. (2016). Air pollution, physical activity, and markers of acute airway oxidative stress and inflammation in adolescents. J. Georg. Public Health Assoc..

[37] Dua S.K., Hopke P.K. (1997). Hygroscopicity of indoor aerosols and its influence on the deposition of inhaled radon decay products. Environ. Int..

[38] Chaney R.A., Sloan C.D., Cooper V.C., Robinson D.R., Hendrickson N.R., McCord T.A., Johnston J.D. (2017). Personal exposure to fine particulate air pollution while commuting: An examination of six transport modes on an urban arterial roadway. PLoS ONE.

[39] Broach J., Bigazzi A.Y. (2017). Existence and Use of Low-Pollution Route Options for Observed Bicycling Trips. Transp. Res. Rec..

[40] Buregeya J.M., Apparicio P., Gelb J. (2020). Short-term impact of traffic-related particulate matter and noise exposure on cardiac function. Int. J. Environ. Res. Public Health.

[41] Pasqua L.A., Damasceno M.V., Cruz R., Matsuda M., Martins M.G., Lima-Silva A.E., Marquezini M., Saldiva P.H.N., Bertuzzi R. (2018). Exercising in air pollution: The cleanest versus dirtiest cities challenge. Int. J. Environ. Res. Public Health.

[42] Carvalho A.M., Krecl P., Targino A.C. (2018). Variations in individuals’ exposure to black carbon particles during their daily activities: A screening study in Brazil. Environ. Sci. Pollut. Res..

[43] Morales Betancourt R., Galvis B., Rincón-Riveros J.M., Rincón-Caro M.A., Rodriguez-Valencia A., Sarmiento O.L. (2019). Personal exposure to air pollutants in a Bus Rapid Transit System: Impact of fleet age and emission standard. Atmos. Environ..

[44] Velasco E., Retama A., Segovia E., Ramos R. (2019). Particle exposure and inhaled dose while commuting by public transport in Mexico City. Atmos. Environ..

[45] Li T., Wang Y., Hou J., Zheng D., Wang G., Hu C., Xu T., Cheng J., Yin W., Mao X. (2019). Associations between inhaled doses of PM2.5-bound polycyclic aromatic hydrocarbons and fractional exhaled nitric oxide. Chemosphere.

[46] Xu T., Hou J., Cheng J., Zhang R., Yin W., Huang C., Zhu X., Chen W., Yuan J. (2018). Estimated individual inhaled dose of fine particles and indicators of lung function: A pilot study among Chinese young adults. Environ. Pollut..

[47] Qiu Z., Wang W., Zheng J., Lv H. (2019). Exposure assessment of cyclists to UFP and PM on urban routes in Xi’an, China. Environ. Pollut..

[48] Lei X., Xiu G., Li B., Zhang K., Zhao M. (2016). Individual exposure of graduate students to PM2.5 and black carbon in Shanghai, China. Environ. Sci. Pollut. Res..

[49] Tan S.H., Roth M., Velasco E. (2017). Particle exposure and inhaled dose during commuting in Singapore. Atmos. Environ..

[50] Jeong H., Park D. (2017). Contribution of time-activity pattern and microenvironment to black carbon (BC) inhalation exposure and potential internal dose among elementary school children. Atmos. Environ..

[51] Manojkumar N., Monishraj M., Srimuruganandam B. (2020). Commuter exposure concentrations and inhalation doses in traffic and residential routes of Vellore city, India. Atmos. Pollut. Res..

[52] Cepeda M., Schoufour J., Freak-Poli R., Koolhaas C.M., Dhana K., Bramer W.M., Franco O.H. (2017). Levels of ambient air pollution according to mode of transport: A systematic review. Lancet Public Health.

[53] Anjilvel Satish Asgharian B. (1995). A Multiple-Path Model of Particle Deposition in the Rat Lung. Toxicol. Sci..

[54] Miller F.J., Asgharian B., Schroeter J.D., Price O. (2016). Improvements and additions to the Multiple Path Particle Dosimetry model. J. Aerosol Sci..

[55] Kuempel E.D., Sweeney L.M., Morris J.B., Jarabek A.M. (2015). Advances in Inhalation Dosimetry Models and Methods for Occupational Risk Assessment and Exposure Limit Derivation. J. Occup. Environ. Hyg..

[56] Hänninen O.O., Alm S., Katsouyanni K., Künzli N., Maroni M., Nieuwenhuijsen M.J., Saarela K., Srám R.J., Zmirou D., Jantunen M.J. (2004). The EXPOLIS study: Implications for exposure research and environmental policy in Europe. J. Expo. Anal. Environ. Epidemiol..

[57] Klepeis N.E., Nelson W.C., Ott W.R., Robinson J.P., Tsang A.M., Switzer P., Behar J.V., Hern S.C., Engelmann W.H. (2001). The National Human Activity Pattern Survey (NHAPS): A resource for assessing exposure to environmental pollutants. J. Expo. Anal. Environ. Epidemiol..

[58] Querol X., Moreno T., Karanasiou A., Reche C., Alastuey A., Viana M., Font O., Gil J., de Miguel E., Capdevila M. (2012). Variability of levels and composition of PM10 and PM2.5 in the Barcelona metro system. Atmos. Chem. Phys..

